# Cold Argon Athmospheric Plasma for Biomedicine: Biological Effects, Applications and Possibilities

**DOI:** 10.3390/antiox11071262

**Published:** 2022-06-27

**Authors:** Andrew K. Martusevich, Alexandra V. Surovegina, Ivan V. Bocharin, Vladimir V. Nazarov, Inessa A. Minenko, Mikhail Yu. Artamonov

**Affiliations:** 1 Laboratory of Translational Free Radical Biomedicine, Sechenov University, 119991 Moscow, Russia; aleksandranikolaeva@icloud.com (A.V.S.); qwer2189@yandex.ru (V.V.N.); akmart@mail.ru (I.A.M.); drmike@mjahealthcare.com (M.Y.A.); 2 MJA Research and Development, Inc., East Stroudsburg, PA 18301, USA; 3 Laboratory of Medical Biophysics, Privolzhsky Research Medical University, 603005 Nizhny Novgorod, Russia; bocharin.ivan@mail.ru; 4 Nizhny Novgorod State Agricultural Academy, 603117 Nizhny Novgorod, Russia; 5 Institute of Applied Physics, 603950 Nizhny Novgorod, Russia

**Keywords:** cold plasma, argon, molecular mechanisms of action, biological effects

## Abstract

Currently, plasma medicine is a synthetic direction that unites the efforts of specialists of various profiles. For the successful formation of plasma medicine, it is necessary to solve a large complex of problems, including creating equipment for generating cold plasma, revealing the biological effects of this effect, as well as identifying and justifying the most promising areas of its application. It is known that these biological effects include antibacterial and antiviral activity, the ability to stimulate hemocoagulation, pro-regenerative properties, etc. The possibility of using the factor in tissue engineering and implantology is also shown. Based on this, the purpose of this review was to form a unified understanding of the biological effects and biomedical applications of argon cold plasma. The review shows that cold plasma, like any other physical and chemical factors, has dose dependence, and the variable parameter in this case is the exposure of its application. One of the significant characteristics determining the specificity of the cold plasma effect is the carrier gas selection. This gas carrier is not just an ionized medium but modulates the response of biosystems to it. Finally, the perception of cold plasma by cellular structures can be carried out by activating a special molecular biosensor, the functioning of which significantly depends on the parameters of the medium (in the field of plasma generation and the cell itself). Further research in this area can open up new prospects for the effective use of cold plasma.

## 1. Introduction

Plasma medicine is a relatively young scientific field, which is the result of successful interdisciplinary interaction of physicists, biologists, and doctors [1,2,3]. Over the past decade, the efforts of researchers from various regions of the world have gradually revealed broad horizons for the use of cold plasma in various fields of medicine. These studies historically originate from technical disciplines within the framework of which the ability of cold plasma to disinfect various surfaces was discovered and investigated. This fact made it possible to extend the scope of application of the physical factor in question to its antibacterial and viricidal activity and, in the future, to other biomedical applications [2,3,4,5]. At the same time, despite the large number of experimental works, their systematization and integration have been carried out partially and only for special areas of application. Insufficient knowledge of the biological effects of cold plasma, as well as their molecular mechanisms, does not allow us to fully test its sanogenetic activity in in vivo models. This determines the fact that most studies are based on the results of in vitro experiments.

Based on this, the purpose of this review was to form a unified understanding of the biological effects and biomedical applications of argon cold plasma.

## 2. Cold Plasma as a Physicochemical Concept

According to the traditional ideas of physicists, plasma is a special, fourth state of matter, along with solids, liquids and gases. Plasma is a partially ionized gas, and the degree of its ionization is one of its key characteristics that determine temperature and other basic parameters and properties. It was the temperature factor that made it possible to divide the plasma into standard (“thermal”), having an average temperature of about 4000–5000 K, and low-temperature (“cold”) with a characteristic temperature of 30–50 °C [1,2,4]. Given the peculiarities of the functioning of living systems, high-temperature plasma cannot be used for medical purposes. On the contrary, low-temperature plasma, causing the generation of reactive oxygen and nitrogen species in solutions and biological objects, can have both positive and negative effects on them [4,6,7,8,9].

In connection with this circumstance, a wide range of generator equipment has been developed using various principles of gas flow ionization. In particular, for this purpose, the authors used spark, arc, barrier, corona discharges, and pulse current, but all these effects provided single process-controlled ionization of the carrier gas [1,4,6]. At the same time, one of the significant difficulties in using these devices is the lack of a configurable variable parameter. On this basis, the gradation of the severity of the action of cold plasma must be carried out only by changing the exposure of the treatment.

## 3. Cold Plasma as a Means of Disinfection

Traditionally, the first biomedical application of cold plasma was the consideration of this physical solution as a method of disinfection and detoxification and decontamination of surfaces. For more than 10 years, there have been reports in the literature about the antibacterial activity of argon cold plasma. One of the most extensive should be considered the study by R. Matthes et al. (2016), in which the resistance of 78 genetically different strains of *Staphylococcus aureus* to the action of cold plasma was studied in detail [10]. It has been shown that the surface properties (in particular, the characteristics of capsule lipopolysaccharide) and antibiotic resistance are associated with resistance to cold plasma treatment. At the same time, a number of pathogens of chronic wound infections (for example, methicillin-resistant strains of *S. aureus* and *Pseudomonas aeruginosa*) were inactivated by exposure to cold plasma [11]. Also of interest are the data of R. Matthes et. al. (2016) on the unequal antibacterial activity of plasma formed from different carrier gases [12]. It was found that argon plasma with 1% oxygen admixture had the greatest effectiveness against staphylococci and pseudomonads. It is important that the presence of oxygen in the medium is more significant than the type of carrier gas [12]. The mechanism of this antibacterial effect is considered to be a toxic effect on the cell wall of microorganisms [13] as well as on the inactivation of key oxygenases of bacteria [14].

Of particular importance is the possibility of eliminating pathogens in biofilms. It is known that such bacterial symbioses significantly complicate the possibility of their destruction by standard antibiotics, whereas the use of cold argon plasma allows for layer-by-layer destruction of biofilms formed by *S. aureus* [15]. Additional ways of antibacterial action of argon cold plasma are the triggering of oxidative stress, DNA damage and the formation of phosphate starvation [16]. This information has been confirmed by other studies involving a wider range of indicators (in particular, the secondary product of lipid peroxidation—malonic dialdehyde) [17].

In addition to the antibacterial activity, the antiviral and fungicidal activity of argon cold plasma flow was later identified and proved. Thus, on a number of bacteriophages (T4, F174 and MS2) it was demonstrated that the considered effect, both in the form of a gas stream and plasma-treated water, successfully inactivates these viral particles [18].

The mechanism of this effect is also based on the oxidative destruction of nucleic acids and proteins. At the same time, a significant increase in the antiviral effect is observed with the additional introduction of 1% of air into the system. These results are fully consistent with the study by H.A. Aboubakr et. al. (2018), who evaluated the ways of inactivation of feline calcivirus with a mixture of 99% argon with 1% oxygen [19]. It was revealed that short-term treatment (15 s) causes only moderate changes in the structure of the virus capsid but complicates its interaction with the infected cell in vitro. Prolonged exposure (2 min) leads to the disintegration of the main capsid proteins, in particular, the main structural domain of the VP1 protein, as well as oxidative damage to viral RNA.

The generation of a massive amount of reactive oxygen species (ROS) also provides a fungicidal effect of argon cold plasma [20,21]. At the same time, this process can be controlled. Thus, according to M.H. Kang et al. (2014), the addition of sodium chloride to the reaction medium significantly reduces the toxic effect of cold plasma on the reproduction, structure and genomic DNA of *Neurospora crassa* [20]. At the same time, the effect of sodium chloride turned out to be slightly more significant than the change in pH, osmolarity, and even the concentration of ROS in the solution under consideration. It can be assumed that such quenching properties of sodium chloride are due to the expenditure of the formed radicals on the generation of hypochlorite (ClO^−^).

On the contrary, the introduction of ROS generation inductors (FeCl2 and FeSO4) into the system, triggering the Fenton reaction, significantly increases the sterilizing properties of argon plasma against *Aureobasidium pullulans* [21]. This fungus produces the black pigment melanin, which ensures its resistance to most damaging effects (up to radiation). Under standard conditions, *A. pullulans* is resistant to cold plasma, but the use of iron salts eliminates this effect, which can be explained by the potentiation of ROS generation in the Fenton reaction [21]. 

Thus, studies of the reaction of microorganisms to cold plasma treatment, on the one hand, are of interest in terms of using the factor as a means of disinfection and antibacterial action. Furthermore, on the other hand, it helps to understand the mechanisms of action of cold plasma on various biological systems.

## 4. Cold Plasma in the Treatment of Biologically Significant Surfaces

An important task of transplantology, dentistry, traumatology and regenerative medicine is the optimal preparation of the implant surface. It is necessary to ensure maximum biocompatibility of these implants, as well as their most complete and successful colonization by cellular elements. It has been shown that pretreatment of the surface of titanium implants with argon cold plasma provides an improvement in the adsorption of bovine serum albumin on them, comparable to the effect of ultraviolet radiation for 2 h [22]. In addition, a series of publications by L. Canullo et al. (2017, 2018) confirmed an improvement in the colonization of scaffolds by osteoblasts after their preconditioning with argon cold plasma due to increased adhesiveness [23,24]. An additional positive characteristic of the effect is the decontamination of the implant surface, and these properties are realized for materials of various compositions [24]. At the same time, stimulation of cell proliferation and cell adhesion to the implant surface is realized in a wide range of exposures (from 5 s to 1.5 min) with equal success [25].

## 5. Non-Thermally Operated Electrosurgical Plasma Sources

Along with the antibacterial activity of cold plasma, procoagulation properties serve as the second earliest discovered property of the ionized gas flow. Thus, the works of M. Zenker (2008) demonstrated the possibility of using thermal plasma flow as an electrosurgery technology that provides hemostasis [26]. The optimal modes of action of the factor were selected, additionally realizing the devitalization of tissues due to thermal exposure. These developments were improved by I. Justin et. al. (2010), who described the design, operation and efficiency of the Cesar 136 brand “plasma knife” operating at a frequency of 13.56 MHz [10]. At the same time, the inner diameter of the blade was about 0.4 mm, which made it possible to achieve high accuracy of the surgical procedure.

The design characteristics of the impact made it possible to successfully apply the “plasma knife” in endoscopic surgery. In particular, within the framework of a randomized pilot clinical trial conducted on patients with Barrett’s esophagus (BRIDGE), the comparability of the results with the effect of radiofrequency ablation (in terms of the effectiveness of removing areas of dysplasia and the frequency of side effects) was demonstrated with a multiple reduction in the cost of the procedure [27].

Currently, the development of cold plasma technologies in surgery has allowed the creation of a number of devices for electrosurgical treatment. In particular, some researchers have shown the possibility of successful application of non-thermal argon plasma in oncogynecology [28,29] and urology [30] based on changes in the state and parameters of the mucosa [29].

## 6. Cold Plasma in Regenerative Medicine and Wound Treatment

The use of ionized inert gas flows in surgery and regenerative medicine is based on two main factors: stimulation of regeneration processes, and antibacterial effects [31,32,33]. The second mechanism is the most studied, as already reported in the relevant section of this review. It should also be noted that both factors are clearly interrelated: the removal of bacterial contamination of the wound creates favorable conditions for adequate restoration of the structure of damaged tissues [11,31,33]. In addition, this is further facilitated by the oxidative removal of necrotic and non-viable tissues under the influence of oxidative stress induced by exposure to cold plasma [31,33]. In this case, the effect of cold plasma therapy will be determined by the exposure of the biological object and the parameters of the plasma itself, primarily by the degree of ionization of the gas stream, which determines the intensity of generation of oxygen, nitrogen radicals and, secondarily, lipids, proteins and nucleic acids. Thus, when using a short exposure (up to 3 min), the bioregulatory effect of the factor is realized, which, in relation to regenerative medicine, manifests itself in stimulating cell proliferation and accelerating the rate of wound healing. This has been demonstrated by various research groups on a wide range of biomodels: from simple (regeneration of the damaged caudal part of the body in Danio fish) [34], to more complex (reproduction of streptozoin-induced diabetic wound [35] and experimental damage to the ear area in rats [36]). All authors note the synergetic, complex effect of cold argon plasma on the regeneration processes and decontamination of pathogenic microorganisms from the wound surface.

Currently, there are some small clinical trials that also prove the effectiveness of the use of the factor in question in the treatment of wounds of various etiologies [32,33]. 

An important additional pathogenetic mechanism of action of cold plasma is a positive effect on the microcirculatory bed. Thus, in our previous studies on the model of contact thermal burn, a pronounced stimulating effect on the intensity of blood flow through small-diameter vessels was established. It should be emphasized that such an effect is provided by physiological regulatory factors—an increase in the release of nitrogen monoxide by the vascular wall, as evidenced by a significant increase in the amplitude of the endothelial component.

In addition, in our experimental studies using the same model, it was demonstrated that exposure to cold plasma induces the generation of reactive oxygen and nitrogen forms, not only in the wound itself and the near-wound zone, but also in the blood of animals. This indicates the presence of a systemic effect of the physical factor in question, which was first established by us both by metabolic (the level of oxidative processes in the blood and various tissues) and functional (the state of heart rate variability and microcirculatory bed in the area remote from the wound) criteria. 

Along with the effectiveness, an important issue determining the possibility of clinical application of cold plasma therapy technology is the safety of the latter. To clarify this, an assessment of the mutagenic effect of argon cold plasma [37] was carried out in modes an order of magnitude higher than the intensity of the standard ones used. The absence of genotoxic effects of the studied factor was shown. A study of the long-term effects of argon cold plasma treatment of the body was also performed. Based on the analysis of the cytokine profile (IL-1, TNF, fetoprotein, calcitonin), as well as the level of cancer-embryonic antigens, there was no increase in the risk of neoplasms one year after the course of cold plasma therapy.

All of the above makes it possible to talk about the relative safety of argon cold plasma; however, additional research is required in this regard.

Finally, the use of prolonged plasma treatment and high-intensity ionization of the gas stream ensure the destructiveness of this factor in relation to biological tissues (“plasma knife technology” [38]) and pathogenic microorganisms.

## 7. Application of Cold Argon Plasma in Oncology

Cold argon plasma can be effectively used for the treatment of tumors of various nature, while its effect can be either direct (directly on tumor structures), or indirect, for example, through plasma-treated aqueous solutions [39,40,41]. It has been shown that the maximum destruction of cancer cells is achieved precisely by using a direct method of exposure [42,43,44]. However, it is worth noting that the location of the tumor is not always available for cold plasma treatment. In these cases, they resort to an indirect method of influence, which has a relatively lower efficiency [40].

The pro-apoptotic effect of cold argon plasma on cancer cells is achieved by generating an excessive amount of long-lived and short-lived reactive oxygen species (ROS) and reactive nitrogen forms (RNS) [43,44,45,46]. In the case of drinking water treatment, stable forms—H_2_O_2_ and NO_2_—have the primary damaging effects [46]. Special attention is paid to the role of glutathione in shifts in the redox status of cells induced by cold argon plasma and resulting from the treatment of prostate cancer [30].

The cellular mechanisms of the proapoptotic effect of argon cold plasma have been studied in detail in experiments in vitro on various tumor cell lines. It has been shown that the treatment of tumor cells causes the launch of apoptotic changes in them. In particular, activation of the caspase system (3, 7, etc.) was detected on osteosarcoma cells, leading to a significant inhibition of the growth of the neoplasm focus as a whole [42]. At the same time, it was found that this effect does not depend on the type of cold plasma generator [42].

Additionally, modulation of the expression of anti-apoptotic genes and stimulation of the production of chaperones—heat shock proteins—are involved in the implementation of programmed tumor cell death induced by the action of cold plasma [43]. Similar results were obtained using the human lymphoma cell line U937, and the effect of cold plasma was enhanced by saturation of the matrix carrier gas with molecular nitrogen, which made it possible to switch the generation of bioradicals from ROS to RNS [43].

In the experiments of another research group (R. Moniruzzaman et al., 2018) performed on Molt-4 lymphoma cells, the fundamental role of hydrogen peroxide formation in ensuring the pro-apoptotic effect of cold plasma on them was established [44]. Hypersynthesis of this molecular messenger determines the activation of the caspase cascade and increases the expression and sensitivity of the FAS receptor to the corresponding ligands [44]. In addition, a valuable result of the work is the proof of the presence of effective modifiers of the damaging effects of the factor in question, in particular, sulfasalazine, which inhibits the cysteine–glutamate antiporter, which provides reduction of the intracellular level of reduced glutathione [44]. This makes it possible to assume the prospects of using this compound as a sensitizer for potentiating the pro-apoptotic effect of cold plasma.

The central role of hydrogen peroxide in the realization of the destructive effects of cold plasma was also confirmed on the A549 lung cancer cell model [39]. Interestingly, during the treatment of cell culture with plasma, a progressive increase in intracellular H_2_O_2_ concentration was recorded, while NO-dependent mechanisms were practically not involved, which was verified by maintaining the level of nitrite ions at the initial values [39]. This highlights the key importance of hydrogen peroxide in the induction of plasma-associated apoptosis of tumor cells.

The involvement of NO-dependent mechanisms in the treatment of tumor cells was demonstrated by the example of the glioblastoma cell line U87MG [40]. It has been shown that in this case, the formation of RNS occurs, which can lead to the development of both apoptosis and necrosis of tumor cells [40,47,48]. It should be noted that the generation of this type of reactive molecules is not necessarily associated with an increase in the concentration of nitrogen in the matrix carrier gas [40]; however, it requires prolonged treatment of a biological object with cold plasma to effectively inhibit cell proliferation and activate cell death [49].

Currently, there are data on the possibility of using cold argon plasma in clinical oncology. Thus, Marzi J. et al. (2022) showed the pilot data of an in-human clinical study using plasma to successfully treat cervical cancer precursors [50]. This allows us to consider oncology as one of the most significant areas of application of cold plasma therapy.

## 8. The Authors’ Own Research in the Field of Studying Local and Systemic Effects of Cold Plasma

Our research attempts to sequentially decipher the biological effects of cold plasma under in vitro and in vivo (on Wistar rats) conditions. In the first stage of our work, ideas about the peculiarities of the action of cold plasma on biosystems of various levels of organization were integrated, and unresolved issues of plasma biomedicine were shown, including deciphering the mechanisms of the biological effect of the factor in question, the effect of the carrier gas on them, the dose dependence of the response of the biological object, etc. [51]. Human whole blood samples served as the main test biosystem, convenient and indicative in terms of evaluating the effects of cold plasma in experiments in vitro. It was found that cold helium plasma modifies various components of blood plasma metabolism [52,53] and erythrocytes [54]. In particular, in blood plasma, the studied factor induced moderate stimulation of free radical processes with a short exposure (1–2 min) against the background of more pronounced activation of the antioxidant system [52]. Similar metabolic shifts have been recorded not only in animals and humans, but also in plants treated with argon cold plasma [55].

It should be emphasized that there was a clear dose dependence of the identified effect on oxidative metabolism. In further studies, it was shown that with an increase in exposure time (in the range of 3 min and more) the degree of activation of free radical oxidation increased progressively [56], which was associated with an avalanche-like increase in the amount of reactive oxygen and nitrogen species, which was confirmed by the data of other authors [4,44,45,48]. These shifts in oxidative metabolism were accompanied by a sharp depletion of the antioxidant reserves of the biological fluid [56].

A similar trend was registered with respect to energy metabolism. According to a number of researchers, the restructuring of this component of metabolism, manifested in shifts in the level of coenzymes (in particular, concentrations of NAD and NADH) and products of these processes (for example, lactate and pyruvate), is due to the regulation of the expression of the corresponding genes (ATP a1, ATP a2, ATP b1, ATP b2, ATP b3, TOR, GRF 1-6) [57].

Interesting features of the response to the action of helium cold plasma were discovered by us when assessing the state of red blood cells. It was found that blood treatment with the studied factor contributed to a pronounced increase in the level of malonic dialdehyde, a standard marker of the intensity of free radical processes, in erythrocytes [56]. Further disclosure of the mechanism of these shifts made it possible to find out that exposure to cold plasma did not lead to stimulation of lipid peroxidation in the membranes of these blood cells, and the detected effect was due to inhibition of aldehyde dehydrogenase utilizing malonic dialdehyde [56]. Subsequently, this pattern was confirmed in experiments in vivo [58].

In addition, the subject of our research was the disclosure of the systemic effects of cold plasma. They were analyzed when exposed to a cold plasma stream on a section of pre-epilated skin of a laboratory animal (Wistar rats). It was found that the treatment under consideration caused nonspecific (adaptive) shifts in heart rate variability [59], as well as stimulation of the intensity of blood flow through small-diameter vessels, realized mainly due to the release of NO by the vascular wall [60]. The conjugacy of the action of cold argon and helium plasma with the modulation of nitric oxide production was previously shown in experimental studies on the model of melanoma [61] and other pathological processes [62], as well as in the mechanisms of the pro-regenerative effect of the studied effect [63]. The systemic nature of the response to the studied physical factor included metabolic rearrangements, which, in particular, were expressed in the activation of energy metabolism enzymes (stimulation of the catalytic properties of lactate dehydrogenase in a direct reaction, accompanied by an increase in pyruvate production) and moderate inhibition of aldehyde dehydrogenase activity [58].

A separate issue is the clarification of the role of the carrier gas in the implementation and features of the action of cold plasma on biological objects. To solve this problem, we compared the modification of the physicochemical properties of blood plasma during its treatment with helium and argon plasma [64]. It was found that the use of helium as a carrier gas provides a more physiological response of the biosystem, contributing to the optimization of the crystalloscopic picture of the biological fluid. On the contrary, the use of argon plasma causes more destructive shifts in the crystallogenesis of blood plasma, but this effect cannot be unequivocally regarded as negative. To a greater extent, it makes it possible to distinguish the areas of practical application of helium and argon cold plasma. We assume that helium plasma mainly has a pro-regenerative effect, while argon plasma has an antibacterial and pro-apoptotic effect. The absence of a negative effect of argon plasma on metabolism was confirmed based on an assessment of the dynamics of the parameters of free radical oxidation and components of the antioxidant system of the blood of rats who received a course of external cold plasma treatment with this carrier gas [65].

In general, the results of our previous studies create prerequisites for further study of the sanogenetic potential of monocomponent cold plasma, primarily antibacterial and regenerative effects, in the treatment of wounds and burns.

## 9. Analysis of Molecular Mechanisms of Cold Plasma Action on Biological Objects

Numerous studies in the field of plasma biomedicine postulate that the cellular, tissue and organismal effects of cold plasma on biosystems are mediated by the formation of reactive oxygen species and nitrogen. Among them, hydrogen peroxide (H_2_O_2_), hydroxyl radical (•OH), ozone (O_3_), singlet oxygen (^1^O_2_), superoxide-anion radical (O_2_^•−^), atomic oxygen (O), nitrogen monoxide (NO), peroxynitrite (•ONOO^−^), nitrite (NO_2_^−^) and others are mentioned [40,66,67]. These compounds are usually classified into short- and long-lived, while the pool of the latter also includes oxidized forms of proteins, DNA and lipoproteins [45,48,66,68,69]. However, for a long time the question of the specific participation of each of these ions has remained open.

**Figure 1:**
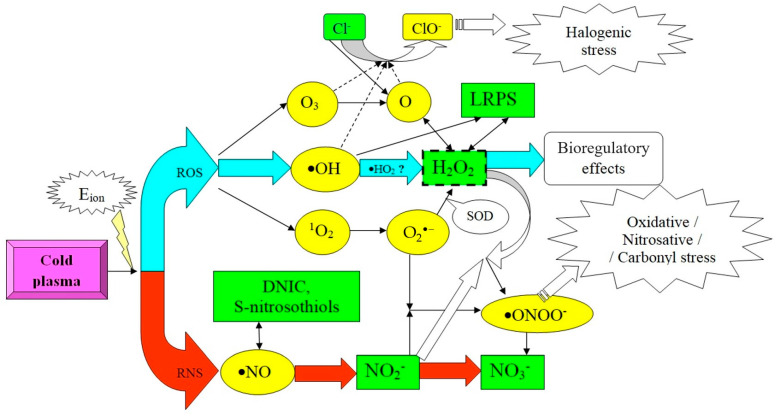
Molecular cascades, induced by the action of cold atmospheric plasma (ROS—reactive oxygen species, RNS—reactive nitrogen species, LRPS—long-living reactive protein species, DNIC—dinitrosyl iron complexes, Eion—ionization energy, SOD—superoxide dismutase).

According to the literature, the central link of the metabolic sensing system–biosensor for cold plasma is hydrogen peroxide, which mediates many other external effects on the cell [39,66,70]. On the other hand, other researchers have reported on the significant role of atomic oxygen [70], singlet oxygen [71], ozone [72], hypochlorite [66] and other reactive molecules in the realization of the effects of cold plasma. Such a diversity of opinions is due, in our opinion, to the emergence of all possible reactive oxygen and nitrogen forms during the processing of biological objects with cold plasma and their integration into a single molecular cascade. The analysis of the physico-chemical foundations of this multifaceted process allowed us to form an integrated circuit of the perception of plasma radiation by the cell and the transduction of this signal to various intracellular structures, including DNA molecules and proteins (Figure 1).

From the presented Figure 1, it can be seen that the effect of cold plasma, which contributes energy to the biosystem for the ionization of endogenous molecules, in a heterogeneous medium leads to the formation of a wide range of reactive oxygen species (the upper branch of the scheme), the main representatives of which in this case are hydroxyl radical, ozone and singlet oxygen. All these compounds are short-lived and are immediately transformed. Thus, ozone decomposes to form atomic oxygen, the hydroxyl radical dimerizes into hydrogen peroxide (as an intermediate stage of this process, the formation of hydroxonium-•NO_2_ can occur), and singlet oxygen forms a superoxide radical. Further, atomic oxygen is capable of oxidizing halides (in particular, Cl^−^) to their oxygen-containing derivatives (ClO^−^, respectively, which can realize halogenating stress) or interact with water to form hydrogen peroxide. Interestingly, the second process can be reversible. It is known that ozone also has the ability to oxygenate halides. In turn, the superoxide anion radical under the action of superoxide dismutase is also transformed into H_2_O_2_. Thus, the entire ROS-dependent cascade of reactions is integrated into a single point—relatively stable hydrogen peroxide. At the same time, long-lived active forms of proteins can act as a kind of depot for this compound. In our opinion, it is hydrogen peroxide that ensures the realization of the bioregulatory effect of cold plasma.

The second (lower section in Figure 1) branch of the molecular sensor for cold plasma is associated with the formation of nitrogen reactive species and is triggered by the generation of the most universal of them—nitric oxide—which can be stored in the form of endogenous depositing compounds (S-nitrosothiols, dinitrosyl iron complexes with various ligands—glutathione, serine or protein [73,74]). The second, more preferable, scenario is the formation of nitrite from NO, which, interacting with a superoxide-anion radical or hydrogen peroxide, provides the synthesis of peroxynitrite, which has an extremely high oxidative potential and probably implements the toxic effects of cold plasma. This creates the conditions for the development of plasma-induced oxidative, nitrous or carbonylating stress (occurs during prolonged exposures of the factor). Finally, both nitrite and peroxynitrite can be transformed into stable and low-reactive nitrate ions, which can be considered as the end point of the process.

It should be noted that the given paths and their preferences depend significantly on the parameters of the environment. In particular, when processing cells in a nitrogen medium, only the NO-dependent branch of the molecular biosensor is involved [43,71]. In the case of additional saturation of the plasma jet with oxygen, its toxic effect is significantly potentiated [19]. A similar effect was achieved when Fe^2+^ ions were introduced into the biosystem, reproducing the Fenton reaction and progressively increasing the concentration of free radicals [21].

On the contrary, the use of various antioxidants or traps of reactive oxygen species (for example, singlet oxygen) partially or completely neutralizes the effect of cold plasma [71]. Additionally, saturation of the system with increased concentrations of sodium chloride reduces the toxic effects of cold plasma even in the case of large exposures [20], which may be due to the “quenching” of the formed reactive oxygen species by chloride ions with the formation of the less reactive hypochlorite. All of the above clearly indicates the controllability of the action of the factor in question through the modulation of the components of the cellular biosensor.

**Figure 2:**
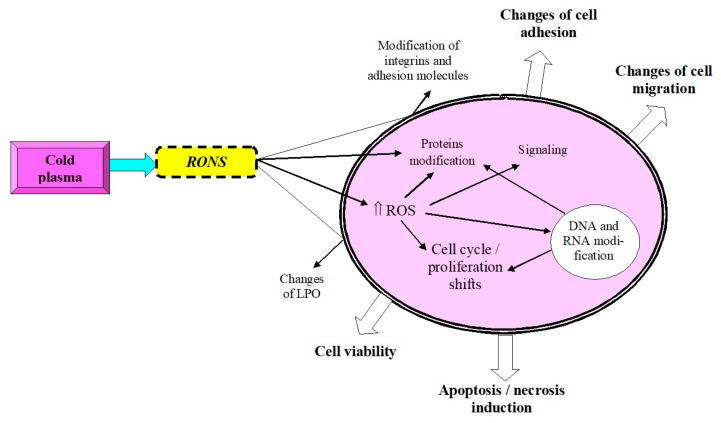
Cellular response to exposure to cold atmospheric plasma (RONS—reactive oxygen and nitrogen species, ROS—reactive oxygen species, LPO—lipid peroxidation).

**Figure 3:**
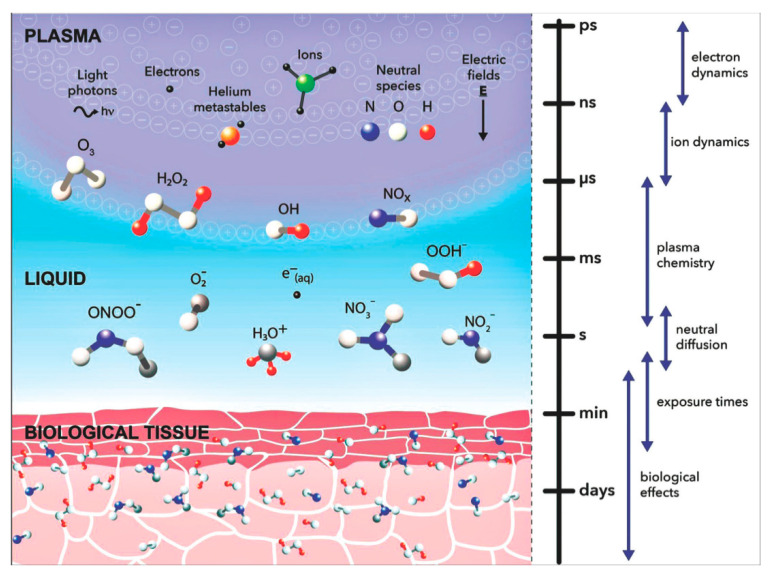
Plasma-induced production of reactive oxygen and nitrogen species in perimembrane space (some data from [9]).

The considered molecular cascades are successfully integrated into the cellular mechanisms of the action of cold plasma on biological objects (Figure 2). Forming reactive oxygen species in the perimembrane space (Figure 3 [75]), cold plasma intensifies their intracellular generation, triggering a metabolic response to the action of this factor [76,77,78,79]. In particular, it is implemented in the oxidative modification of proteins and nucleic acids, as well as in the regulation of the functioning of cell organelles. In addition, changes in the cell cycle and the processes of proliferation and differentiation can occur [57,80,81]. The mechanism of these effects may be due to the effect on plasma proteins (hemoglobin, myoglobin, lysozyme, etc.) [82], as well as with the involvement of p53 protein modulators [83].

A study of plasma–3D–tissue interactions showed tissue penetration depth of physical plasma in human mucosa [84]. The authors used marker-independent Raman spectroscopy to determine the functional tissue penetration depth of direct plasma treatment. A working group at the NMI (Natural and Medical Science Institute) published data comparing the analysis of protein expression using Digi-West technology following plasma treatment of 2D single cells and 3D tissue [85]. This study shows the importance of investigating plasma effects on 3D tissue due to significantly differing results compared to single cell studies, which so far represent the (incomplete) data basis of our understanding of plasma effects. Holl M. et al. (2022) showed that in the culture of peritoneal cells, such effects can be caused not only by direct irradiation with cold plasma, but also by plasma-activated water [86]. Interestingly, this liquid form of exposure to cold plasma can be used as a drink. In particular, it has been shown that oral administration of plasma-activated water for 28 days can inhibit proliferation and angiogenesis, as well as stimulate apoptosis in a model of non-small cell lung cancer in mice [87].

The rearrangement of the cell membrane caused by the modification of the peroxidation of its structural lipids and proteins in combination with conformational and oxidative shifts of integrins and adhesion molecules can lead either to stabilization of the plasmalemma or to its desiccation [88,89]. The latter serves as a mechanism for the implementation of plasma-induced apoptosis (in some cases, with prolonged exposure to the action of the necrosis factor). Additionally, these shifts in the membrane state can significantly change the adhesive properties of the cell and affect its migration [90,91,92]. 

Yan et al. suggested that an increased density of aquaporins in the membrane of tumor cells facilitates the entry of plasma-derived ROS and RNS into tumor cells and thus causes selective apoptosis induction [93]. The model by Van der Paal et al. is focusing on the role of cholesterol for the control of ROS influx into cells [94]. These two models are based on the concept that plasma-derived ROS and RNS are sufficient to induce apoptosis. This is contrasted by a model presented by Bauer and Graves that is based on an initial signaling effect of singlet oxygen, either derived from plasma directly or formed through the interaction between long-lived ROS and RNS with the extracellular redox system on the surface of tumor cells. This may trigger singlet oxygen formation by tumor cells and induction of NADPH oxidase 1 (NOX1)-driven and ROS- and RNS-mediated apoptosis [95].

Thus, the treatment of biological objects with cold plasma, activating specific molecular mechanisms, ensures the transformation of the functioning of cells and tissues.

## 10. Conclusions

In general, plasma biomedicine currently appears to be a multifaceted interdisciplinary field that has already demonstrated broad prospects in various fields of medicine. Like any other physico-chemical factors, cold plasma is dose-dependent, and the variable parameter is the exposure of its application. One of the significant parameters determining the specificity of the cold plasma effect is the carrier gas used, which is not just an ionized medium, but modulated the response of biosystems to it. Finally, the perception of cold plasma by cellular structures can be carried out by activating a special molecular biosensor, the functioning of which significantly depends on the parameters of the medium (in the field of plasma generation and the cell itself). Further research in this area may open up new prospects for the effective use of cold plasma.

In our opinion, an in-depth understanding of the biological effects of argon cold plasma, as well as the molecular mechanisms of their implementation, makes it possible to extend the scope of this method. In particular, it becomes possible to use cold plasma more widely as a means of stimulating regenerative processes, as well as a modulator of the state of the microcirculatory bed. In addition, the disclosure of the systemic effects of cold plasma makes it possible to correct the functional and metabolic status of the body with a wide range of pathology. Thus, cold plasma therapy can be considered not only as a method of local treatment, but also as a systemic medical technology.

## References

[1] Laroussi M. (2020). Cold plasma in medicine and healthcare: The new frontier in low temperature plasma applications. Front. Phys..

[2] Von Woedtke T., Schmidt A., Bekeschus S., Wende K., Weltmann K.-D. (2019). Plasma medicine: A field of applied redox biology. In Vivo.

[3] von Woedtke T., Emmert S., Metelmann H.R., Rupf S., Weltmann K.D. (2020). Perspectives on cold atmospheric plasma (CAP) applications in medicine. Phys. Plasmas.

[4] Lotfy K. (2016). Cold atmospheric plasma and oxidative stress: Reactive oxygen species vs. antioxidant. Austin Biochem..

[5] Scholtz V., Pazlarova J., Souskova H., Khun J., Julak J. (2015). Nonthermal plasma—A tool for decontamination and disinfection. Biotechnol. Adv..

[6] Kong M.G., Kroesen G., Morfill G., Nosenko T., Shimizu T., Van Dijk J., Zimmermann J.L. (2009). Plasma medicine: An introductory review. New J. Phys..

[7] Hoffmann C., Berganza C., Zhang J. (2013). Cold atmospheric plasma: Methods of production and application in dentistry and oncology. Med. Gas Res..

[8] Yan D., Sherman J.H., Keidar M. (2017). Cold atmospheric plasma, a novel promising anti-cancer treatment modality. Oncotarget.

[9] Dubuc A., Monsarrat P., Virard F., Merbahi N., Sarrette J.P., Laurencin-Dalicieux S., Cousty S. (2018). Use of cold-atmospheric plasma in oncology: A concise systematic review. Ther. Adv. Med. Oncol..

[10] Justan I., Tichý F., Slavícek P. (2010). A new type of plasma knife and its effect on biological issue—A pilot study. Acta Chir. Plast..

[11] Mohd Nasir N., Lee B.K., Yap S.S., Thong K.L., Yap S.L. (2016). Cold plasma inactivation of chronic wound bacteria. Arch. Biochem. Biophys..

[12] Matthes R., Bekeschus S., Bender C., Koban I., Hübner N.O., Kramer A. (2012). Pilot-study on the influence of carrier gas and plasma application (open resp. delimited) modifications on physical plasma and its antimicrobial effect against *Pseudomonas aeruginosa* and *Staphylococcus aureus*. GMS Krankenhhyg. Interdiszip..

[13] Guo J., Li Z., Huang K., Li Y., Wang J. (2017). Morphology analysis of *Escherichia coli* treated with nonthermal plasma. J. Appl. Microbiol..

[14] Todorova Y., Yotinov I., Topalova Y., Benova E., Marinova P., Tsonev I., Bogdanov T. (2019). Evaluation of the effect of cold atmospheric plasma on oxygenases’ activities for application in water treatment technologies. Environ. Technol..

[15] Traba C., Liang J.F. (2015). The inactivation of *Staphylococcus aureus* biofilms using low-power argon plasma in a layer-by-layer approach. Biofouling.

[16] Winter T., Bernhardt J., Winter J., Mäder U., Schlüter R., Weltmann K.D., Kusch H. (2013). Common versus noble *Bacillus subtilis* differentially responds to air and argon gas plasma. Proteomics.

[17] Hosseinzadeh Colagar A., Memariani H., Sohbatzadeh F., Valinataj Omran A. (2013). Nonthermal atmospheric argon plasma jet effects on *Escherichia coli* biomacromolecules. Appl. Biochem. Biotechnol..

[18] Guo L., Xu R., Gou L., Liu Z., Zhao Y., Liu D., Kong M.G. (2018). Mechanism of Virus Inactivation by Cold Atmospheric-Pressure Plasma and Plasma-Activated Water. Appl. Environ. Microbiol..

[19] Aboubakr H.A., Mor S.K., Higgins L., Armien A., Youssef M.M., Bruggeman P.J., Goyal S.M. (2018). Cold argon-oxygen plasma species oxidize and disintegrate capsid protein of feline calicivirus. PLoS ONE.

[20] Kang M.H., Hong Y.J., Attri P., Sim G.B., Lee G.J., Panngom K., Park G. (2014). Analysis of the antimicrobial effects of nonthermal plasma on fungal spores in ionic solutions. Free Radic. Biol. Med..

[21] Fukuda S., Kawasaki Y., Izawa S. (2019). Ferrous chloride and ferrous sulfate improve the fungicidal efficacy of cold atmospheric argon plasma on melanized *Aureobasidium pullulans*. J. Biosci. Bioeng..

[22] Canullo L., Genova T., Tallarico M., Gautier G., Mussano F., Botticelli D. (2016). Plasma of Argon Affects the Earliest Biological Response of Different Implant Surfaces. An In Vitro Comparative Study. J. Dent. Res..

[23] Canullo L., Genova T., Naenni N., Nakajima Y., Masuda K., Mussano F. (2018). Plasma of argon enhances the adhesion of murine osteoblasts on different graft materials. Ann. Anat..

[24] Canullo L., Genova T., Wang H.L., Carossa S., Mussano F. (2017). Plasma of Argon Increases Cell Attachment and Bacterial Decontamination on Different Implant Surfaces. Int. J. Oral. Maxillofac. Implant..

[25] Seon G.M., Seo H.J., Kwon S.Y., Lee M.H., Kwon B.J., Kim M.S., Park J.C. (2015). Titanium surface modification by using microwave-induced argon plasma in various conditions to enhance osteoblast biocompatibility. Biomater. Res..

[26] Zenker M. (2008). Argon plasma coagulation. GMS Krankenhhyg. Interdiszip..

[27] Peerally M.F., Bhandari P., Ragunath K., Barr H., Stokes C., Haidry R., de Caestecker J.S. (2019). Radiofrequency ablation compared with argon plasma coagulation after endoscopic resection of high-grade dysplasia or stage T1 adenocarcinoma in Barrett’s esophagus: A randomized pilot study (BRIDE). Gastrointest. Endosc..

[28] Feil L., Koch A., Utz R., Ackermann M., Barz J., Stope M., Krämer B., Wallwiener D., Brucker S.Y., Weiss M. (2020). Cancer-Selective Treatment of Cancerous and Non-Cancerous Human Cervical Cell Models by a Non-Thermally Operated Electrosurgical Argon Plasma Device. Cancers.

[29] Wenzel T., Carvajal Berrio D.A., Reisenauer C., Layland S., Koch A., Wallwiener D., Weiss M. (2020). Trans-Mucosal Efficacy of Non-Thermal Plasma Treatment on Cervical Cancer Tissue and Human Cervix Uteri by a Next Generation Electrosurgical Argon Plasma Device. Cancer.

[30] Weiss M., Gümbel D., Hanschmann E.-M., Mandelkow R., Gelbrich N., Zimmermann U., Walther R., Ekkernkamp A., Sckell A., Kramer A. (2015). Cold Atmospheric Plasma Treatment Induces Anti-Proliferative Effects in Prostate Cancer Cells by Redox and Apoptotic Signaling Pathways. PLoS ONE.

[31] Salehi S., Shokri A., Khani M.R., Bigdeli M., Shokri B. (2015). Investigating effects of atmospheric-pressure plasma on the process of wound healing. Biointerphases.

[32] Bekeschus S., Schmidt A., Niessner F., Gerling T., Weltmann K.D., Wende K. (2017). Basic Research in Plasma Medicine—A Throughput Approach from Liquids to Cells. J. Vis. Exp..

[33] Haertel B., Woedtke T. (2014). von, Weltmann, K.D.; Lindequist, U. Non-thermal atmospheric-pressure plasma possible application in wound healing. Biomol. Ther..

[34] Nam M.K., Kim G.Y., Yun S.E., Jang J.Y., Kim Y.H., Choi E.H., Rhim H. (2017). Harmless effects of argon plasma on caudal fin regeneration and embryogenesis of zebrafish: Novel biological approaches for safe medical applications of bioplasma. Exp. Mol. Med..

[35] Cheng K.Y., Lin Z.H., Cheng Y.P., Chiu H.Y., Yeh N.L., Wu T.K., Wu J.S. (2018). Wound Healing in Streptozotocin-Induced Diabetic Rats Using Atmospheric-Pressure Argon Plasma Jet. Sci Rep..

[36] Schmidt A., von Woedtke T., Stenzel J., Lindner T., Polei S., Vollmar B., Bekeschus S. (2017). One Year Follow-Up Risk Assessment in SKH-1 Mice and Wounds Treated with an Argon Plasma Jet. Int. J. Mol. Sci..

[37] Kluge S., Bekeschus S., Bender C., Benkhai H., Sckell A., Below H., Kramer A. (2016). Investigating the Mutagenicity of a Cold Argon-Plasma Jet in an HET-MN Model. PLoS ONE.

[38] Matthes R., Lührman A., Holtfreter S., Kolata J., Radke D., Hübner N.O., Kramer A. (2016). Antibacterial Activity of Cold Atmospheric Pressure Argon Plasma against 78 Genetically Different (mecA, luk-P, agr or Capsular Polysaccharide Type) *Staphylococcus aureus* Strains. Skin Pharmacol. Physiol..

[39] Jo A., Joh H.M., Chung T.H., Chung J.W. (2020). Anticancer Effects of Plasma-Activated Medium Produced by a Microwave-Excited Atmospheric Pressure Argon Plasma Jet. Oxid. Med. Cell Longev..

[40] Chen Z., Simonyan H., Cheng X., Gjika E., Lin L., Canady J., Keidar M. (2017). A novel micro cold atmospheric plasma device for glioblastoma both in vitro and in vivo. Cancers.

[41] Keidar M. (2015). Plasma for cancer treatment. Plasma Sour. Sci. Technol..

[42] Haralambiev L., Wien L., Gelbrich N., Lange J., Bakir S., Kramer A., Stope M.B. (2020). Cold atmospheric plasma inhibits the growth of osteosarcoma cells by inducing apoptosis, independent of the device used. Oncol. Lett..

[43] Tabuchi Y., Uchiyama H., Zhao Q.L., Yunoki T., Andocs G., Nojima N., Kondo T. (2016). Effects of nitrogen on the apoptosis of and changes in gene expression in human lymphoma U937 cells exposed to argon-based cold atmospheric pressure plasma. Int. J. Mol. Med..

[44] Moniruzzaman R., Rehman M.U., Zhao Q.L., Jawaid P., Mitsuhashi Y., Imaue S., Noguchi M. (2018). Roles of intracellular and extracellular ROS formation in apoptosis induced by cold atmospheric helium plasma and X-irradiation in the presence of sulfasalazine. Free Radic. Biol. Med..

[45] Graves D.B. (2012). The emerging role of reactive oxygen and nitrogen species in redox biology and some implications for plasma applications to medicine and biology. J. Phys. D Appl. Phys..

[46] Tian W., Kushner M.J. (2014). Atmospheric pressure dielectric barrier discharges interacting with liquid covered tissue. J. Phys. D Appl. Phys..

[47] Boehm D., Heslin C., Culler P.J., Bourke P. (2016). Cytotoxic and mutagenic potential of solutions exposed to cold atmospheric plasma. Sci. Rep..

[48] Kim S.J., Chung T. (2016). Cold atmospheric plasma jet-generated RONS and their selective effects on normal and carcinoma cells. Sci. Rep..

[49] Wende K., Straßenburg S., Haertel B., Harms M., Holtz S., Barton A., Lindequist U. (2014). Atmospheric pressure plasma jet treatment evokes transient oxidative stress in HaCaT keratinocytes and influences cell physiology. Cell Biol. Int..

[50] Marzi J., Stope M.B., Henes M., Koch A., Wenzel T., Holl M., Layland S.L., Neis F., Bösmüller H., Ruoff F. (2022). Noninvasive Physical Plasma as Innovative and Tissue-Preserving Therapy for Women Positive for Cervical Intraepithelial Neoplasia. Cancers.

[51] Martusevich A.K., Kostrov A.V. (2018). Biomedical applications of microwave radiation: Innovative approaches. EPJ Web of Conferences.

[52] Martusevich A.K., Soloveva A.G., Yanin D.V., Galka A.G., Krasnova S.Y. (2017). The effect of helium cold plasma on the parameters of oxidative blood metabolism in vitro. Bull. New Med. Technol..

[53] Martusevich A.K., Galka A.G., Golygina E.S. (2020). Modifying the blood’s physical and chemical parameters using cold helium plasma: In vitro study. Plasma Med..

[54] Martusevich A.K., Solov’eva A.G., Galka A.G., Kozlova L.A., Yanin D.V. (2018). Effects of Helium Cold Plasma on Erythrocyte Metabolism. Bull. Exp. Biol. Med..

[55] Kim S.Y., Lee S.Y., Min S.C. (2019). Improvement of the Antioxidant Activity, Water Solubility, and Dispersion Stability of Prickly Pear Cactus Fruit Extracts Using Argon Cold Plasma Treatment. J. Food Sci..

[56] Martusevich A.K., Galka A.G., Karuzin K.A., Tuzhilkin A.N., Malinovskaya S.L. (2021). Cold helium plasma as a modifier of free radical processes in the blood: In vitro study. AIMS Biophys..

[57] Zhang J.J., Jo J.O., Huynh D.L., Mongre R.K., Ghosh M., Singh A.K., Lee S.B., Mok Y.S., Hyuk P., Jeong D.K. (2017). Growth-inducing effects of argon plasma on soybean sprouts via the regulation of demethylation levels of energy metabolism-related genes. Sci. Rep..

[58] Martusevich A.K., Soloveva A.G., Krasnova S.Y., Galka A.G., Kostrov A.V. (2020). Effect of cold helium plasma on the catalytic activity of certain erythrocyte dehydrogenases of rat blood. Proceedings of Universities. Appl. Chem. Biotechnol..

[59] Martusevich A.K., Krasnova S.Y., Peretyagin P.V., Galka A.G., Golygina E.S., Kostrov A.V. (2019). The effect of heli-um-generated cold plasma on the parameters of heart rate variability in rats. Biophysics.

[60] Martusevich A.K., Krasnova S.Y., Galka A.G., Peretyagin P.V., Yanin D.V., Kostrov A.V. (2019). Estimation of the micro-circulatory response to the effect of cold helium plasma. Biophysics.

[61] Alimohammadi M., Golpur M., Sohbatzadeh F., Hadavi S., Bekeschus S., Niaki H.A., Valadan R., Rafiei A. (2020). Cold Atmospheric Plasma Is a Potent Tool to Improve Chemotherapy in Melanoma In Vitro and In Vivo. Biomolecules.

[62] Lackmann J.W., Bruno G., Jablonowski H., Kogelheide F., Offerhaus B., Held J., Schulz-von der Gathen V., Stapelmann K., von Woedtke T., Wende K. (2019). Nitrosylation vs. oxidation—How to modulate cold physical plasmas for biological applications. PLoS ONE.

[63] García-Alcantara E., López-Callejas R., Morales-Ramírez P.R., Peña-Eguiluz R., Fajardo-Muñoz R., Mercado-Cabrera A., Barocio S.R., Valencia-Alvarado R., Rodríguez-Méndez B.G., Muñoz-Castro A.E. (2013). Accelerated mice skin acute wound healing in vivo by combined treatment of argon and helium plasma needle. Arch. Med. Res..

[64] Martusevich A.K., Galka A.G., Golygina E.S., Fedotova A.S., Tuzhilkin A.N., Malinovskaya S.L. (2021). Comparative Study of the Influence of Helium and Argon Plasma on Crystallogenic Properties of the Blood. Plasma Med..

[65] Martusevich A.K., Karuzin K.A., Nazarov V.V., Malinovskaya S.L. (2021). Effect of cold helium plasma on oxidative metabolism and crystallogenic properties of rat blood. Int. J. Plasma Environ. Sci. Technol..

[66] Bekeschus S., Wende K., Hefny M.M., Rödder K., Jablonowski H., Schmidt A., Benedikt J. (2017). Oxygen atoms are critical in rendering THP-1 leukaemia cells susceptible to cold physical plasma-induced apoptosis. Sci. Rep..

[67] Rehman M.U., Jawaid P., Uchiyama H., Kondo T. (2016). Comparison of free radicals formation induced by cold atmospheric plasma, ultrasound, and ionizing radiation. Arch. Biochem. Biophys..

[68] Gebicki S., Gebicki J.M. (1999). Crosslinking of DNA and proteins induced by protein hydroperoxides. Biochem. J..

[69] Kim K.C., Piao M.J., Madduma Hewage S.R.K., Han X.I.A., Kang K.A., Jo J.O., Hyun J.W. (2016). Non-thermal dielectric-barrier discharge plasma damages human keratinocytes by inducing oxidative stress. Int. J. Mol. Med..

[70] Uchiyama H., Zhao Q.L., Hassan M.A., Andocs G., Nojima N., Takeda K., Kondo T. (2015). EPR-Spin Trapping and Flow Cytometric Studies of Free Radicals Generated Using Cold Atmospheric Argon Plasma and X-Ray Irradiation in Aqueous Solutions and Intracellular Milieu. PLoS ONE.

[71] Schmidt A., Bekeschus S., Jablonowski H., Barton A., Weltmann K.D., Wende K. (2017). Role of Ambient Gas Composition on Cold Physical Plasma-Elicited Cell Signaling in Keratinocytes. Biophys. J..

[72] Wende K., Williams P., Dalluge J., Van Gaens W., Aboubakr H., Bischof J., Bruggeman P.J. (2015). Identification of the biologically active liquid chemistry induced by a nonthermal atmospheric pressure plasma jet. Biointerphases.

[73] Suryo Rahmanto Y., Kalinowski D.S., Lane D.J., Lok H.C., Richardson V., Richardson D.R. (2012). Nitrogen monoxide (NO) storage and transport by dinitrosyl-dithiol-iron complexes: Long-lived NO that is trafficked by interacting proteins. J. Biol. Chem..

[74] Vanin A.F. (2009). Dinitrosyl iron complexes with thiolate ligands: Physico-chemistry, biochemistry and physiology. Nitric. Oxide Biol. Chem..

[75] Hirst A.M., Frame F.M., Arya M., Maitland N.J., O’Connell D. (2016). Low temperature plasmas as emerging cancer therapeutics: The state of play and thoughts for the future. Tumour. Biol..

[76] Privat-Maldonado A., Schmidt A., Lin A., Weltmann K.D., Wende K., Bogaerts A., Bekeschus S. (2019). ROS from Physical Plasmas: Redox Chemistry for Biomedical Therapy. Oxid. Med. Cell Longev..

[77] Chandana L., Sangeetha C.J., Shashidhar T., Subrahmanyam C. (2018). Non-thermal atmospheric pressure plasma jet for the bac-terial inactivation in an aqueous medium. Sci. Total Environ..

[78] Blackert S., Haertel B., Wende K., von Woedtke T., Lindequist U. (2013). Influence of non-thermal atmospheric pressure plasma on cellular structures and processes in human keratinocytes (HaCaT). J. Dermatol. Sci..

[79] Choi J.H., Song Y.S., Lee H.J., Kim G.C., Hong J.W. (2017). The topical application of low-temperature argon plasma enhances the anti-inflammatory effect of Jaun-ointment on DNCB-induced NC/Nga mice. BMC Complement. Altern. Med..

[80] Feng X., Ma X., Liu H., Xie J., He C., Fan R. (2019). Argon plasma effects on maize: Pesticide degradation and quality changes. J. Sci. Food Agric..

[81] Haertel B., Straßenburg S., Oehmigen K., Wende K., von Woedtke T., Lindequist U. (2013). Differential influence of components resulting from atmospheric-pressure plasma on integrin expression of human HaCaT keratinocytes. Biomed. Res. Int..

[82] Park J.H., Kim M., Shiratani M., Cho A.E., Choi E.H., Attri P. (2016). Variation in structure of proteins by adjusting reactive oxygen and nitrogen species generated from dielectric barrier discharge jet. Sci. Rep..

[83] Attri P., Kurita H., Koga K. (2021). and Shiratani, M. Impact of Reactive Oxygen and Nitrogen Species Produced by Plasma on Mdm2–p53 Complex. Int. J. Mol. Sci..

[84] Wenzel T., Carvajal Berrio D.A., Daum R., Reisenauer C., Weltmann K.D., Wallwiener D., Brucker S.Y., Schenke-Layland K., Brauchle E.M., Weiss M. (2019). Molecular Effects and Tissue Penetration Depth of Physical Plasma in Human Mucosa Analyzed by Contact- and Marker-Independent Raman Microspectroscopy. ACS Appl. Mater. Interfaces.

[85] Ruoff F., Henes M., Templin M., Enderle M., Bösmüller H., Wallwiener D., Brucker S.Y., Schenke-Layland K., Weiss M. (2021). Targeted Protein Profiling of In Vivo NIPP-Treated Tissues Using DigiWest Technology. Appl. Sci..

[86] Holl M., Rasch M.-L., Becker L., Keller A.-L., Schultze-Rhonhof L., Ruoff F., Templin M., Keller S., Neis F., Keßler F. (2022). Cell Type-Specific Anti-Adhesion Properties of Peritoneal Cell Treatment with Plasma-Activated Media (PAM). Biomedicines.

[87] Song C.-H., Attri P., Ku S.-K., Han I., Bogaerts A., Choi E.H. (2021). Cocktail of reactive species generated by cold atmospheric plasma: Oral administration induces non-small cell lung cancer cell death. J. Phys. D Appl. Phys..

[88] Kim H., Kim H.J., Kim H.K., Hong J.Y., Cho S.B. (2020). Effects of argon and nitrogen plasma pulses on the skin and skin appendages in an in vivo animal model. Skin Res. Technol..

[89] Schmidt A., Wende K., Bekeschus S., Bundscherer L., Barton A., Ottmüller K., Masur K. (2013). Non-thermal plasma treatment is associated with changes in transcriptome of human epithelial skin cells. Free Radic. Res..

[90] Reitberger H.H., Czugala M., Chow C., Mohr A., Burkovski A., Gruenert A.K., Fuchsluger T.A. (2018). Argon Cold Plasma-A Novel Tool to Treat Therapy-resistant Corneal Infections. Am. J. Ophthalmol..

[91] Canady J., Wiley K., Ravo B. (2006). Argon plasma coagulation and the future applications for dual-mode endoscopic probes. Rev. Gastroenterol. Disord..

[92] Nomura T., Miyashita M., Makino H., Maruyama H., Katsuta M., Kashiwabara M., Tajiri T. (2007). Argon plasma coagulation for the treatment of superficial esophageal carcinoma. J. Nippon Med. Sch..

[93] Yan D., Talbot A., Nourmohammadi N.T., Sherman J.H., Cheng X., Keidar M. (2015). Toward understanding the selective anticancer capacity of cold atmospheric plasma—A model based on aquaporins (Review). Biointerphases.

[94] Kubinova S., Zaviskova K., Uherkova L., Zablotskii V., Churpita O., Lunov O., Dejneka A. (2017). Non-thermal air plasma promotes the healing of acute skin wounds in rats. Sci. Rep..

[95] Bauer G., Graves D.B. (2016). Mechanisms of Selective Antitumor Action of Cold Atmospheric Plasma-Derived Reactive Oxygen and Nitrogen Species. Plasma Process. Polym..

